# Long-term stability of RNA in post-mortem bovine skeletal muscle, liver and subcutaneous adipose tissues

**DOI:** 10.1186/1471-2199-8-108

**Published:** 2007-11-29

**Authors:** Bojlul Bahar, Frank J Monahan, Aidan P Moloney, Olaf Schmidt, David E MacHugh, Torres Sweeney

**Affiliations:** 1 UCD School of Agriculture, Food Science and Veterinary Medicine, University College Dublin, Belfield, Dublin 4, Ireland; 2 UCD School of Biology and Environmental Science, University College Dublin, Belfield, Dublin 4, Ireland; 3 Teagasc, Grange Beef Research Centre, Dunsany, Co. Meath, Ireland

## Abstract

**Background:**

Recovering high quality intact RNA from post-mortem tissue is of major concern for gene expression studies in animals and humans. Since the availability of post-mortem tissue is often associated with substantial delay, it is important that we understand the temporal variation in the stability of total RNA and of individual gene transcripts so as to be able to appropriately interpret the data generated from such studies. Hence, the objective of this experiment was to qualitatively and quantitatively assess the integrity of total and messenger RNA extracted from bovine skeletal muscle, subcutaneous adipose tissue and liver stored at 4°C at a range of time points up to 22 days post-mortem. These conditions were designed to mimic the environment prevailing during the transport of beef from the abattoir to retail outlets.

**Results:**

The 28S and 18S rRNA molecules of total RNA were intact for up to 24 h post-mortem in liver and adipose tissues and up to 8 days post-mortem in skeletal muscle. The mRNA of housekeeping genes (*GAPDH *and *ACTB*) and two diet-related genes (*RBP5 *and *SCD*) were detectable up to 22 days post-mortem in skeletal muscle. While the mRNA stability of the two housekeeping genes was different in skeletal muscle and liver, they were similar to each other in adipose tissue. After 22 days post-mortem, the relative abundance of *RBP5 *gene was increased in skeletal muscle and in adipose tissue and decreased in liver. During this period, the relative abundance of *SCD *gene also increased in skeletal muscle whereas it decreased in both adipose tissue and liver.

**Conclusion:**

Stability of RNA in three tissues (skeletal muscle, subcutaneous adipose tissue and liver) subjected to long-term post-mortem storage at refrigeration temperature indicated that skeletal muscle can be a suitable tissue for recovering biologically useful RNA for gene expression studies even if the tissue is subjected to post-mortem storage for weeks, whereas adipose tissue and liver should be processed within 24 hours post-mortem.

## Background

High quality intact RNA is an essential requirement for gene expression studies. The extent of RNA degradation is of major concern for studies analysing tissues that have been subjected to a long post-mortem delay. Recent studies have suggested that the stability of RNA in post-mortem tissue varies with the type of tissue and the post-mortem tissue storage conditions [1-4]. Several studies also suggested that even a considerable delay in processing of tissues may not adversely affect the RNA integrity as reflected in the abundance of specific messenger RNA (mRNA) transcripts [1,5]. In the bovine, in spite of the potential application of RNA technology in investigating the underlying biochemical pathways of determining post-mortem meat quality, meat authentication and forensics, the feasibility of recovering intact RNA from tissues from commercial meat plant where availability of tissue samples often involve a time lag due to carcass processing, or from meat samples after a long post-mortem delay such as those from a retail market chain, has not been investigated. Since the post-mortem stability of an mRNA population can not be generalised [6,7], knowledge of tissue specific stability of mRNA transcripts of interest is essential for evaluating the usefulness of post-mortem tissues for gene expression studies.

Gene expression profiling holds great potential for unravelling complex biological traits in livestock including the interaction of dietary nutrients in gene expression [8-10] and the downstream implications on meat quality attributes [11,12]. Although knowledge of real time expression of genes in the live animal can only be generated through functional analysis of biologically active tissues ideally obtained through biopsy, gene expression studies frequently need to be conducted on post-mortem tissues, which may often be subjected to various lengths of post-mortem delay [11]. For example, gene expression studies in bovine tissues undergoing long post-mortem delay are often inevitable for studying the post-mortem changes determining meat quality traits like tenderness, flavour and juiciness [11].

With the availability of the bovine genome sequence database and the advent of commercially available microarray and SNP-chips, the role of novel candidate gene/genes in animal health, nutrition and physiology are being investigated. Animal nutri-genomic studies involving the expression of genes in animal tissues due to the action of dietary nutrients has potential applications in developing gene expression based bio-markers for livestock production and authentication [9,10,12]. Genes whose expression can be influenced by dietary components from different production systems of livestock like the retinol binding protein gene 5 (*RBP5*) [13,14] and the stearoyl-CoA desaturase gene (*SCD*) [15] in cattle, have the potential to serve as bio-markers of different production systems. The expression of the *RBP5 *gene in the bovine tissue is likely to reflect the dietary intake of β-carotene, which is abundant in fresh grasses [16]. Similarly, expression of the *SCD *gene occurs due to dietary inclusion of feedstuffs rich in essential fatty acids derived from natural sources such as fresh grasses and plant and fish oils [17]. Consequently, the expression levels of these two genes have the potential to serve as biomarkers for grass-based production systems in bovine tissues.

The tissue specific variation of RNA stability in laboratory animals and humans subjected to various length of post-mortem time has been extensively investigated in forensics [18,19] and human disease diagnosis [20-22]. However, in the bovine, the potential of recovering biologically useful RNA remains unexplored. RNA stability during post-mortem storage of animal tissues in general and of bovine tissues consumed as meat in particular, remains mostly unknown since time-points beyond 96 h were not investigated in previous studies. It is a normal practice for beef to be consumed after 2–3 weeks due to the fact that an elaborate meat tenderization process occurs over this time period. This experiment was designed to mimic the real life situation (tissue processing, temperature and time) from the slaughtering of the animal to the length of time when meat is made available to consumers in retail outlets. The objective of this experiment was to qualitatively and quantitatively assess the integrity of total and mRNA extracted from bovine skeletal muscle, subcutaneous adipose tissue and liver stored at 4°C at a range of time points up to 22 days post-mortem. These tissue storage conditions were designed to mimic the environment prevailing during the transport of beef from the abattoir to retail outlets.

## Results

### Stability of total RNA in skeletal muscle

**Figure 1:**
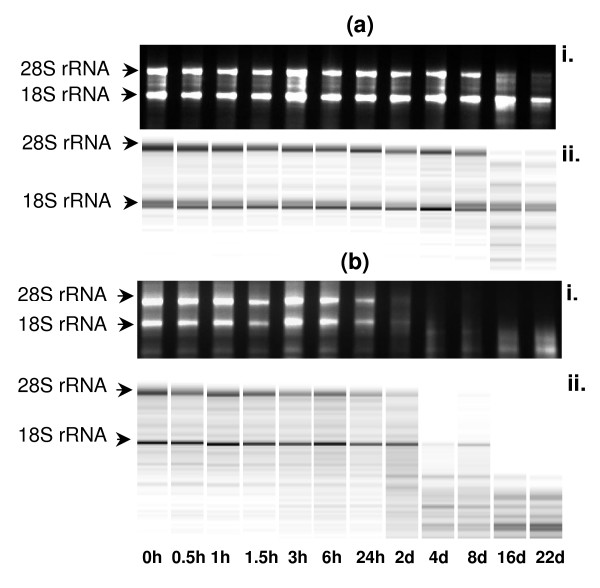
**Stability of total RNA in bovine tissues during post-mortem storage at 4°C**. The stability of total RNA was determined by agarose gel electrophoresis assay (i) and Agilent Bioanalyzer assay in (ii) skeletal muscle (a) and liver (b) (h- hours; d- days at 4°C). (For subcutaneous adipose tissue, because of the presence of high variability among the four animals studied, it was not appropriate to select an individual representative animal.)

A similar total RNA stability pattern was observed in all four animals used in this study in skeletal muscle samples. Intact 28S ribosomal RNA (rRNA) was apparent up to 8 days and 18S rRNA up to 22 days post-mortem as determined by agarose gel electrophoresis (Figure 1). This was consistent with the banding pattern generated with the Agilent Bioanalyzer. The appearance of conspicuous low molecular weight bands at 16 days post-mortem indicated that some RNA degradation had occurred after 8 days post-mortem (Figure 1).

**Figure 2:**
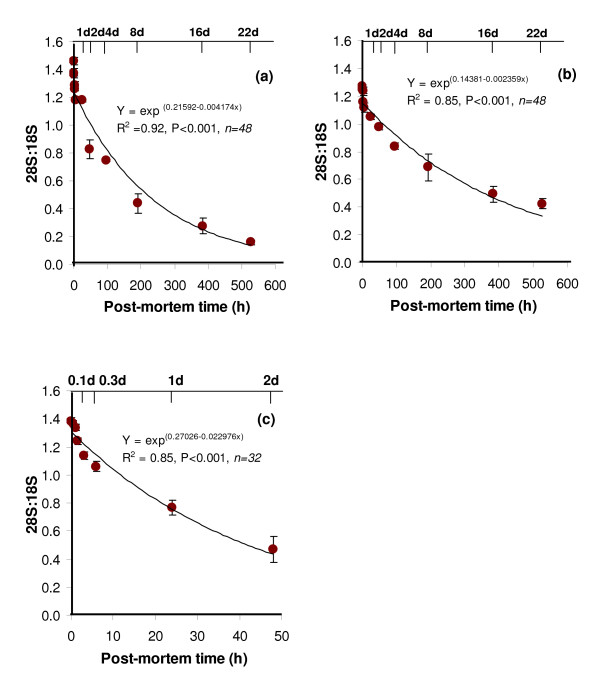
**Change in the 28S:18S ratio of total RNA in bovine tissues during post-mortem storage at 4°C**. The 28S:18S ratio was determined by Agilent Bioanalyzer for bovine skeletal muscle (a), subcutaneous adipose tissue (b) and liver (c) (error bar = 1 SE of mean, n = 4). *Note: the axis has been modified for liver to 2 d*.

The rate of change of the intensity of the two major rRNA molecules over 22 days post-mortem was determined by exponential regression analysis (Table 1). The high molecular weight rRNA (28S) was less stable than low molecular weight rRNA (18S) during post-mortem storage. The pattern of change in the ratio of the 28S:18S rRNA over the post-mortem time period, as calculated by the exponential regression analysis, indicated that the 28S:18S ratio decreased by 50% after 6.9 days post-mortem (Figure 2).

**Table 1:** Rate of degradation of 28S and 18S rRNA in bovine skeletal muscle, adipose tissue and liver and the statistics of the slope from the exponential regression model

	Half-life	*R*^2^	*P <*	Slope	SE
**28S rRNA**					
Skeletal muscle *(48)*	141 h (5.9 d)	*0.91*	0.001	-0.00491	0.00023
Adipose tissue *(48)*	125 h (5.2 d)	*0.66*	0.001	-0.00557	0.00060
Liver *(32)*	21 h (0.9 d)	*0.75*	0.001	-0.03309	0.00347
**18S rRNA**					
Skeletal muscle*	-	-	-	-	-
Adipose tissue *(48)*	216 h (9.0 d)	*0.45*	0.001	-0.00321	0.00053
Liver *(32)*	69 h (2.9 d)	*0.19*	0.05	-0.01009	0.00376

(numbers in parenthesis is the n = number of observation used for half-life calculation, *due to the non-significant (P > 0.05) change in the intensity of 18S rRNA of the skeletal muscle after 22 days post-mortem, exponential model failed to fit the 18S data)

### Stability of mRNA in skeletal muscle

**Figure 3:**
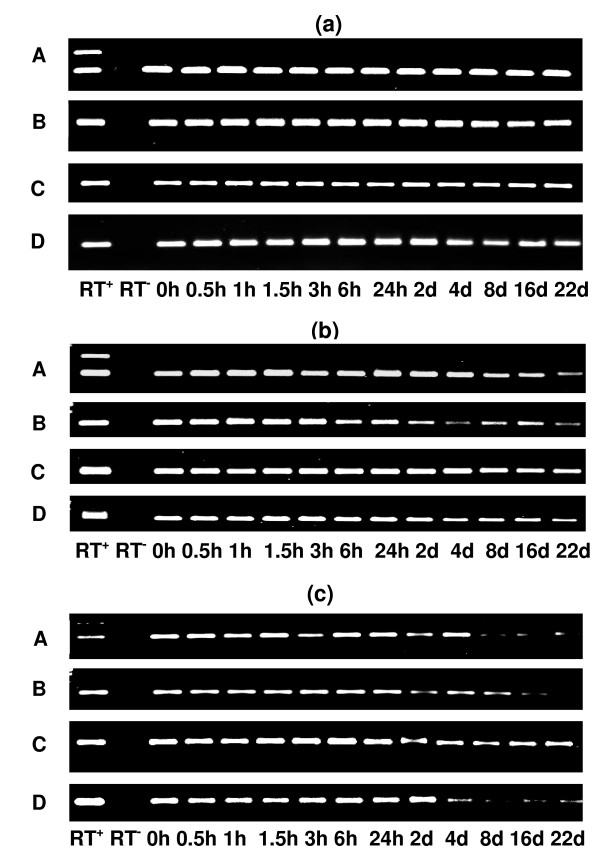
**Agarose gel electrophoresis of RT-PCR products from bovine tissues subjected to post-mortem storage at 4°C up to 22 days**. The amplification products from gene specific RT-PCR as assayed on 1.5% agarose gel for skeletal muscle (a), subcutaneous adipose tissue (b) and liver (c) (h- hours; d- days at 4°C). A: *GAPDH*, B: *ACTB*, C: *RBP5*, D: *SCD*. RT^+^: positive control with genomic DNA, RT^-^: negative control with no template DNA.

The Reverse Transcriptase - Polymerase Chain Reaction (RT-PCR) products of two housekeeping genes (*GAPDH *and *ACTB*) and two diet-related genes (*RBP5 *and *SCD*) were detectable on agarose gels and had uniform band intensities over the 22 days post-mortem (Figure 3). The real time quantitative RT-PCR (qRT-PCR) data were analysed by *geNorm *and *Norm Finder *(see Materials and methods for details) to measure the overall stability of *GAPDH*, *ACTB*, *RBP5 *and *SCD *gene transcripts in post-mortem skeletal muscle (Table 2). Both methods assigned the highest stability (characterised by the *M *value of *geNorm *and the stability value of *Norm Finder*) to *ACTB *among the four genes. On the other hand, *RBP5 *gene was the most unstable in post-mortem skeletal muscle. The stability ranking of *GAPDH *and *SCD *genes slightly differed between the two methods of analysis.

**Table 2:** Overall stability of *GAPDH*, *ACTB*, *RBP5 *and *SCD *genes in post-mortem bovine skeletal muscle (S/Muscle), adipose tissue and liver up to 22 days post-mortem. Analysis was performed by using *geNorm *and *Norm Finder*.

	*geNorm (stability factor M)*			*Norm Finder (stability value)*		
		
Genes	S/Muscle	Adipose	Liver	S/Muscle	Adipose	Liver
*GAPDH*	1.40	2.58	0.69	0.58	0.86	0.19
*ACTB*	1.15	2.52	0.94	0.38	0.78	0.26
*RBP5*	1.67	3.04	0.68	0.85	1.17	0.18
*SCD*	1.36	2.80	0.70	0.63	1.07	0.19

**Figure 4:**
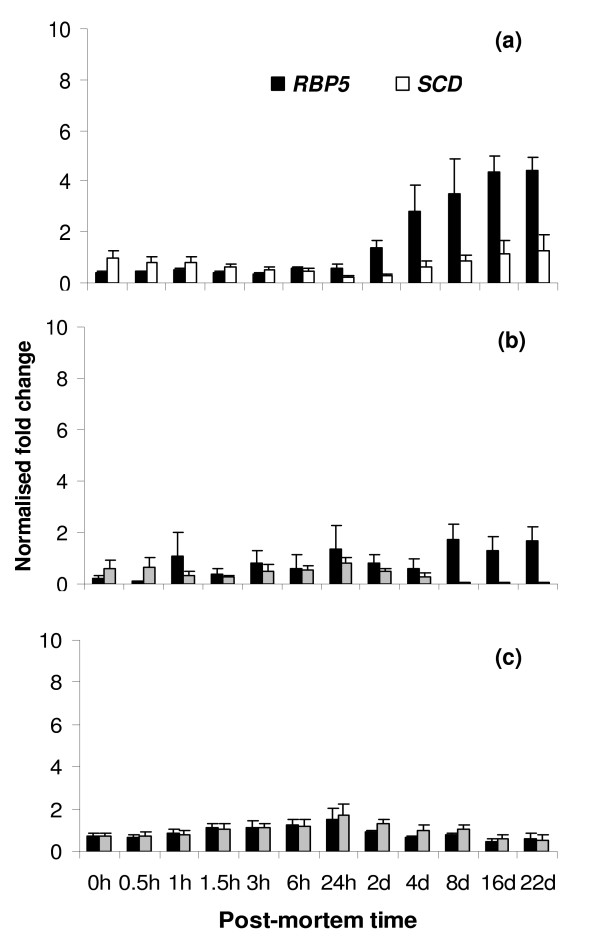
**Normalised relative abundance of *RBP5 *and *SCD *gene transcripts in bovine tissues during post-mortem storage at 4°C**. Normalization was performed relative to the housekeeping gene/genes in skeletal muscle (a), subcutaneous adipose tissue (b) and liver (c) (error bar = 1 SE of mean (n = 4)).

The normalised relative abundance of *RBP5 *and *SCD *gene transcripts in post-mortem skeletal muscle clearly showed that compared to 0 h time-point, after 22 days post-mortem, there was an increase in the relative abundances of *RBP5 *and *SCD *genes (Figure 4). The relative abundance pattern of these two genes indicated that deterioration of total RNA quality as characterised by disappearance of 28S rRNA after 8 days post-mortem (Figure 1) had no adverse affect on the relative abundances of these two genes.

### Stability of total RNA in adipose tissue

The 28S and 18S rRNA molecules were intact at 24 h post-mortem in adipose tissue in all animals. However, there was a high intra- and inter-animal variability in the intensities of the two rRNA bands on agarose gel (data not included in fig 1 because of the lack of an individual representative animal). The RNA stability profile of the four animals indicated that after 24 h post-mortem, there was substantial variation in the degradation of the 28S rRNA. Although the 28S rRNA degraded more quickly than 18S rRNA in post-mortem adipose tissue, degradation of the latter was also evident during 22 days of post-mortem storage (Table 1). The pattern of change in the ratio of the 28S:18S rRNA over the post-mortem time period, as calculated by the exponential regression analysis, indicated that the 28S:18S ratio decreased by 50% after 11.7 days post-mortem (Figure 2).

### Stability of mRNA in subcutaneous adipose tissue

The RT-PCR products of the *GAPDH, ACTB, RBP5 *and *SCD *genes were visible on agarose gels (Figure 3), although the band intensities gradually decreased over time for all genes with the exception of *RBP5*. The observed intra animal variability of gene transcripts as characterised by slightly variable band intensities across the time points may likely be due to the characteristics of adipose tissue samples which caused intra animal variability of total RNA stability. Adipose tissue characteristics such as different thickness of subcutaneous adipose tissue used for sampling at different time points could be a contributing factor to this intra animal variability. As analysed by *geNorm*, the stability of *GAPDH *and *ACTB *genes were closely similar to each other in post-mortem adipose tissue, however, slightly higher stability was assigned to the *ACTB *gene compared to the *GAPDH *gene when the same data were analysed by *Norm Finder *(Table 2). The *RBP5 *gene was the most unstable of the four gene transcripts evaluated in post-mortem adipose tissue (this instability value can be associated with an increase or decrease in the relative abundance of a gene). Compared to the 0 h time-point, the normalised relative abundance of the *RBP5 *gene increased whereas that of the *SCD *gene decreased over the 22 days post-mortem (Figure 4).

### Stability of total RNA in liver

A rapid decline in the intensities of the 28S and 18S rRNA bands after 24 h post-mortem indicated that RNA was unstable in post-mortem liver (Figure 1). The 28S and 18S rRNA degraded substantially after 24 h and 48 h post-mortem, respectively. The 28S rRNA was not detectable after 48 h using the Agilent Bioanalyzer; therefore, quantification of total RNA degradation in liver was performed only up to this time-point (n = 32). Short half-lives of both rRNA molecules in post-mortem liver were evident (Table 1). The 28S:18S ratio decreased by 50% after 30 h post-mortem (Figure 2).

### Stability of mRNA in liver

The agarose gel electrophoresis of RT-PCR products indicated that the gene transcripts of *GAPDH*, *ACTB *and *SCD *genes rapidly decreased over time in post-mortem liver (Figure 3). However, the amplification was obtained up to 22 days post-mortem for the *RBP5 *gene. Somewhat variable degrees of amplification also occurred at the later time points for *GAPDH*, *ACTB *and *SCD *genes which could be due to amplification of the gene transcript using too many (40) PCR amplification cycles. The overall stability of the gene transcripts, as determined by *geNorm *and *Norm Finder*, indicated that *GAPDH*, *RBP5 *and *SCD *genes had closely similar stability and that the *ACTB *gene was the most unstable in post-mortem liver. The pattern of change in the relative abundances of *RBP5 *and *SCD *genes showed a rapid decline after 24 h post-mortem (Figure 4).

## Discussion

The present study evaluated the qualitative and quantitative variations in the integrity of total RNA and mRNA of two common housekeeping genes (*GAPDH *and *ACTB*) and two diet-related genes (*RBP5 *and *SCD*) in bovine skeletal muscle, adipose tissue and liver stored at 4°C for up to 22 days post-slaughter. The tissue storage conditions were designed to mimic the environment prevailing during the transportation of beef from the abattoir to retail outlets. Tissue specific variation existed in the stability of 28S and 18S rRNA in post-mortem bovine tissues. Total RNA was more stable in skeletal muscle compared to both adipose tissue and liver. Similarly, real time RT-PCR analysis in these tissues revealed the presence of gene specific variation in the stability of mRNA transcripts of *GAPDH, ACTB, RBP5 *and *SCD *genes.

The most surprising result of this study was the fact that total RNA was present for 8 days and specific mRNA transcripts were present for as long as 22 days post-mortem in skeletal muscle. In contrast, the RNA transcripts degraded much more quickly in liver and adipose tissues. To our knowledge, there have been no previous investigations on the stability of total RNA or specific gene transcripts in any biological tissue over such a long time period. Previous studies have observed intact total RNA in bovine reproductive tissues up to 24 h post-mortem at room temperature [2] and in connective tissues and liver of rabbit up to 96 h post-mortem at 4°C [1]. RNA stability at longer post-mortem times is unclear because time-points beyond 96 h were unreported in previous studies.

The underlying cellular mechanism that permits the rRNA molecules to remain intact for a considerably long period of time in post-mortem bovine muscle at 4°C has not been ascertained. However, since the storage temperature, post-mortem pH and metabolic activeness of tissue can greatly influence the integrity of RNA [4,23], the high stability of RNA in skeletal muscle could be due to a cellular microenvironment that is unfavourable to rapid degradation of RNA molecules. Skeletal muscle tissue is unusual in terms of its molecular activity post-mortem, as a series of biochemical pathways are actively involved in the meat tenderisation process [24,25]. In contrast, the low stability of total RNA in liver may be due to some ribonuclease activity at 4°C since liver is a ribonuclease rich tissue *in vivo *[26,27]. In fact, highly unstable RNA in mammalian liver has been previously reported [1,3]. The high inter-animal variation of RNA stability in adipose tissue observed in this study may be due to the variable thickness of the subcutaneous adipose tissue layers whereby cellular nucleases, induced by the mechanical injury, may be associated in thin adipose tissue layers.

Changes in the relative abundance of different mRNA transcripts were observed throughout the 22-day experimental time period. For instance, the stability of the two housekeeping genes (both *geNorm *and *Norm Finder *gave consistent results) was different in skeletal muscle with *ACTB *more stable than *GAPDH*. Variation in mRNA stability has previously been observed in other mammalian tissues though *GAPDH *mRNA was more stable than *ACTB *in bovine uterine tissues [2] and similar to each other in rat brain tissue [3]. The suitability of RNA collected post-mortem for the expression of specific genes is ultimately determined by the presence of intact mRNA populations containing the mRNA transcripts of interest. Even in the later collections of tissue where there was considerable 28S and 18S rRNA degradation, mRNA transcripts were still present for the diet-related genes of interest. This is in agreement with the findings previously been reported in micro-array hybridization [7] and real time qRT-PCR assays [27]. Recent findings on gene specific stability revealed wide variation among different species of mRNA [7,28]. The stability of any specific mRNA is highly influenced both by the primary and secondary structures of the transcript and the rate of translation *in vivo *[29]. The implication of this variation in RNA stability over time is that it would not be appropriate to perform microarray experiments on these tissues and extrapolate the data back to studies of global gene expression in the live animal [30]. It would however, be of interest to identify the gene clusters and systems pathways that are still active during this time period as they are most likely fundamentally involved in biochemical pathways that influence parameters such as meat tenderisation, flavour etc.

The two major rRNA molecules had somewhat variable stability where the 18S had higher stability compared to 28S in all three tissues. Higher stability of 18S rRNA molecules has been previously reported [1,4]. Although the 28S:18S rRNA ratio is a commonly used measure of total RNA integrity [27,30], our data suggests that the 28S:18S ratio is not a reliable marker for evaluating the suitability of tissue samples for gene expression analysis, especially when the 18S rRNA also degrades. For example, it was observed that 28S:18S rRNA ratio decreased from 1.3 (0 h time point) to 0.4 (22 days time point) in adipose tissue, whereas the same decreased from 1.5 (0 h time point) to 0.2 (22 days time point) in skeletal muscle. In the latter instance, the 18S rRNA was highly stable and the decrease in 28S:18S was mostly due to a decrease in the intensity of 28S rRNA molecules. Unlike in skeletal muscle, in adipose tissue, as the 28S, the intensity of 18S rRNA also decreased over time and due to this decrease (both numerator and denominator decrease), the 28S:18S ratio still remain high even after 22 days post-mortem. Therefore, 28S:18S rRNA ratio could be misleading in situations where both the rRNA molecules degrade proportionately.

Interestingly, the stability measurements indicated that *RBP5 *mRNA was least stable in skeletal muscle and adipose tissues although there were substantial increases in the relative abundances of this gene in these two tissues up to 22 days post-mortem. This further showed that in these two tissues, the 28S:18S ratio of RNA inadequately reflects the presence of specific mRNA transcripts. Whether the post-mortem relative abundance of the *RBP5 *mRNA was due to a high stability of the mRNA transcript or a continuous transcription of the gene, needs further research. Recent work involving mRNA stability of post-mortem mammalian tissues has shown that there are a subset of mRNA that may increase with increasing post-mortem storage time [7,28] which is in agreement with the observed high abundance of the *RBP5 *mRNA. Unlike skeletal muscle, the *RBP5 *mRNA in liver degraded quickly. Since mRNA turnover is high in liver, the degradation of the majority of mRNA species in post-mortem liver may occur quickly. In skeletal muscle, the *SCD *mRNA was also considerably unstable since the relative abundance of *SCD *gene considerably increased after 22 days post-mortem. The *SCD *mRNA was least stable in the adipose tissue but the reason is unclear. A number of factors, including the cellular content of fatty acids, may influence the stability of *SCD *mRNA as demonstrated in adipocyte culture [31,32].

## Conclusion

The results of this experiment demonstrate that RNA is more stable in post-mortem skeletal muscle at refrigeration temperature than adipose tissue and liver. Quantitative gene expression analysis by real time RT-PCR showed that the relative abundance of gene transcripts in post-mortem tissues varies with the type of tissue and gene species. The skeletal muscle can be a suitable tissue for recovering biologically useful RNA even if the tissue is subjected to post-mortem storage for weeks whereas adipose tissue and liver should be processed within 24 hours post-mortem.

## Methods

### Experimental animals

Four heifers (75% Limousin breed) at an approximately 20 months of age and initial body weight of 599.3 ± 36.8 kg were fed *ad libitum *pasture grasses for 16 weeks. The animals also received a barley-based concentrate diet containing 15% (on a fresh weight basis) of sunflower oil at 2 kg d^-1 ^per animal. These diets were chosen as the *RBP5 *gene should be expressed as a result of the dietary β-carotene in the pasture grasses and the *SCD *should be expressed as a result of the fatty acids contained in the sunflower oil and pasture grasses, respectively.

### Collection and storage of tissues

Animals were slaughtered in accordance with EU approved guidelines for animal welfare and standard procedures. The point at which bleeding of the animals was initiated was considered time zero post-mortem (0 h) and the 0 h muscle (*M. Longissimus dorsi*) and subcutaneous adipose tissue samples were collected within 2 min of the initiation of bleeding, using a sterile knife pre-treated with RNase AWAY^® ^(Sigma-Aldrich Corp. St. Louis, MO, USA). The 0 h liver sample was taken within 4 min of the initiation of bleeding.

Following hide removal, further muscle, adipose tissue and liver samples (2–3 g for each tissue) were taken at 0.5, 1.0, 1.5, 3.0, 6.0 and 24 h post-mortem. The muscle and adipose tissue samples were taken at approx. 15 cm intervals along the length of the *M. Longissimus thoracis et lumborum *(LTL). The tissue sampling took place in tandem with the normal evisceration and carcass veterinary inspection processes. The LTL was dissected from each carcass following the 24 h sampling and cut into 20 cm sections that were vacuum packed, stored at 4°C and sampled at 2, 4, 8, 16 and 22 days post-mortem. Sampling of the excised liver (which was vacuum packaged and stored at 4°C), took place at the same time points as for muscle and adipose tissue. All samples were snap frozen in liquid N and then stored at -86°C prior to RNA extraction.

### Extraction and analysis of RNA

Total RNA was extracted from muscle (100 mg), liver (40 mg) and adipose tissue (100 mg) with TRI Reagent^® ^(Molecular Research Centre, Inc. USA) according to the manufacturer's instructions. Extracted RNA was dissolved in 20 μl of 0.1% DEPC treated water and then subjected to deoxyribonuclease I (DNase I) (Qiagen, Chatsworth, CA, USA). Re-extraction of RNA was carried out using phenol-chloroform. The RNA pellet was finally dissolved in 20 μl of nuclease-free water.

Total RNA (1 μl/sample) was visualised on a 1.2% agarose gel. The purity of total RNA was evaluated on a spectrophotometer (Ultrospec 2000, Pharmacia Biotech, Cambridge, UK) and samples with a 260/280 ratio ≥ 2.0 were used for further analysis. The quality and quantity of total RNA were assayed by analysing 1 μl of total RNA on an Agilent 2100 Bioanalyzer version A.02.12 (Agilent Technologies, Inc. CA, USA) using RNA Nano LabChips^® ^(Caliper Technologies Corp. MA, USA).

### cDNA synthesis

The cDNA synthesis was performed using oligo(dT)_20 _primer with 1.0 μg of the total RNA in a final reaction volume of 20 μl using the Superscript™ III First-strand synthesis system for reverse transcriptase-polymerase chain reaction (RT-PCR) (Invitrogen Corp., San Diego, CA, USA) following the manufacturer's protocol. At the final step of the cDNA synthesis reaction, *E. coli *RNase H (Invitrogen Corp.) treatment was performed to digest all RNA including the mRNA template resulting in the production of single stranded cDNA to serve as a template in the subsequent PCR reaction.

### RT-PCR analysis

Due to the tissue-specific expression of the retinol binding protein (*RBP*) gene family, one member of the family (*RBP5*), which is expressed in mammalian muscle, adipose tissue and liver [33], was chosen. A basic local alignment search tool (BLAST) homology search with the human *RBPIII *gene sequence was performed and this procedure identified the ortholog *RBP5 *sequence of the bovine [GeneBank: XM_587041]. The expression of the bovine *RBP5 *mRNA was then validated in bovine muscle, adipose tissue and liver by RT-PCR analysis.

Oligonucleotide primers were designed with Primer Express^® ^version 2.0 (Applied Biosystems, Foster City, CA, USA) (Table 3). The RT-PCR amplification was carried out in a PTC-225 DNA Engine Tetrad (MJ Research, Inc. Massachusetts, USA) with 0.5 μl cDNA template in a 25 μl final reaction volume with 1 × PCR buffer, 0.2 μM dNTP mix, 1.5 mM MgCl_2, _0.2 μM of each of the forward and reverse primers, and 0.5 unit of *Taq *DNA polymerase (Invitrogen Corp.). The PCR cycle conditions were 94°C for 30 sec, 60°C for 30 sec and 72°C for 30 sec for forty cycles followed by a final extension at 72°C for 10 min for all genes. Positive (50 ng bovine genomic DNA) and negative template controls (no template) were run in parallel as RT-PCR quality control checks. PCR products were visualised on 1.5% agarose gels.

**Table 3:** Nucleotide sequences of primers and the PCR product size of *GAPDH, ACTB, RBP5 *and *SCD *genes

Gene	Primer sequence	Product (bp)
**GAPDH**	Forward 5'GCATCGTGGAGGGACTTATGA 3'	67
[GeneBank:U85042]	Reverse 5'GGGCCATCCACAGTCTTCTG 3'	
**ACTB**	Forward 5'CGCCATGGATGATGATATTGC 3'	66
[GeneBank:AY141970]	Reverse 5'AAGCGGCCTTGCACAT 3'	
**RBP5**	Forward 5'GACGCAAATGCCAGACCATA 3'	51
[GeneBank:XM_587041]	Reverse 5'CACAAACCAGCTGCTCCTCTT 3'	
**SCD**	Forward 5'GCTGCTTGTGCGCAAACA 3'	72
[GeneBank:AB075020]	Reverse 5'TCGGCTCTTAGGTCGGATAAATT 3'	
	Probe 5'CAGCTGTCAAAGAAAAGGGTTCCACGCT 3'	

In this experiment, gene expression studies were performed using one sets of primer per gene. The use of oligo(dT)_20 _primer for cDNA synthesis ensured a stringent selection of mRNA populations with intact 3' polyA tails, and therefore partially degraded mRNA with shorter polyA tails would be excluded. Due to the fact that initial cDNA templates used in the gene specific amplification in PCR were intact and the PCR efficiency of the primer pairs were high (> 80%), it may be expected that the quantitative data on the relative abundance of the mRNA transcripts generated by use of single sets of primer per gene would be similar to those generating through the use of different sets of primers targeting different regions of the same gene template. The presence of two bands in the genomic DNA (used as positive template control for the RT-PCR) and one band in the cDNA for the *GAPDH *gene provided an effective screen of the cDNA samples for genomic DNA contamination.

### Real time quantitative RT-PCR (qRT- PCR)

The relative abundances of the gene transcripts were determined using a 7900 HT Sequence Detection System (Applied Biosystems). For the *GAPDH*, *ACTB *and *RBP5 *genes, amplification was performed using a 10 μl reaction mixture containing 0.5 μl of cDNA solution, 100–200 nM each of the forward and reverse primers and 1 × SYBR^® ^Green PCR master mix (Invitrogen Corp.). For the *SCD *gene, amplifications were performed as above, with the addition of 100 nM of probe (Table 3) and 1 × TaqMan^® ^PCR master mix (Applied Biosystems). The thermal cycle conditions were 94°C for 30 sec followed by 60°C for 1 min, for 40 cycles, followed by dissociation of the product at 96°C for 2 min. Three non-template controls were analysed in parallel for every real time qRT-PCR run. The mRNA abundances were expressed in *C*_t _values, the number of PCR cycles after which the PCR product crosses a threshold value [34]. Each sample was run in triplicate.

### Statistical analysis

The intensities of 28S and18S rRNA measured by the Bioanalyzer and their ratio in the total RNA over the post-mortem time were analysed by exponential regression analysis. From the slope of the least square exponential regression, the half-life of rRNA molecules were calculated.

Since the amount of tissue used for RNA extraction varied in muscle, adipose and liver, normalisation of the *C*_t _values obtained from real-time RT-PCR was performed independently for each tissue. Normalisation was performed by i) transforming the raw *C*_t _values to relative quantities using the formula, relative quantities = (PCR efficiency) ^Δ*C*t^, where Δ*C*_t _is the change in the *C*_t _values of the sample relative to the highest expression (minimum *C*_t _value) at 0 h time point. ii) normalization was performed relative to the most stable housekeeping gene in skeletal muscle and liver. For adipose tissue, since the two housekeeping genes had similar stability, the geometric mean of the relative quantities of the two housekeeping genes were used for normalization [35]. The normalised fold change or the relative abundance of each of the *RBP5 *and *SCD *genes were calculated by dividing their relative quantities by the normalisation factor. The overall stability of *GAPDH, ACTB, RBP5 *and *SCD *gene mRNA in skeletal muscle, adipose tissue and liver was evaluated following two excel based programmes *geNorm *[35] and *Norm Finder *[36]. For both programmes, raw *C*_t _values were first transformed into the relative quantities using the formula stated above.

## Authors' contributions

BB performed all the experimental steps and was the primary author of the manuscript. FM, OS and DM were involved in designing the experiment and AM in supervising the animal feeding. TS participated in the designing of the study and supervising the RNA analysis. All authors actively participated in the preparing and reviewing the manuscript.
